# Artificial Intelligence in Healthcare Administration and Clinical Informatics: A Critical Review and Governance Roadmap

**DOI:** 10.3390/healthcare14111497

**Published:** 2026-05-28

**Authors:** Hanadi Aldosari

**Affiliations:** Department of Artificial Intelligence and Data Science, College of Computer Science and Engineering, Taibah University, Medina 42353, Saudi Arabia; hdosari@taibahu.edu.sa

**Keywords:** artificial intelligence, medical informatics, healthcare administration, clinical decision support, electronic health records, AI governance, interoperability, healthcare equity

## Abstract

Artificial intelligence (AI) is increasingly influencing healthcare administration and clinical informatics by supporting disease diagnosis, clinical decision-making, treatment personalization, drug discovery, remote monitoring, public health surveillance, and hospital operations. However, the successful adoption of AI in healthcare depends not only on algorithmic performance, but also on its safe integration into clinical information systems, organizational workflows, and governance structures. This article presents a narrative critical review of recent advances in AI-driven healthcare, with a focus on four major domains: AI-enabled disease diagnosis, treatment personalization and clinical decision support, drug discovery and biomedical knowledge generation, and healthcare administration. Evidence from radiology, pathology, ophthalmology, dermatology, and cardiology shows that AI systems can achieve strong diagnostic performance in selected settings, while applications in electronic health records, natural language processing, telemedicine, and predictive analytics are increasingly used to support healthcare delivery and operational decision-making. At the same time, important barriers continue to limit real-world implementation, including fragmented data infrastructures, limited interoperability, poor data quality, algorithmic bias, lack of explainability, privacy and cybersecurity risks, unclear accountability, and insufficient external validation. This review critically examines these challenges and proposes a governance-oriented roadmap for responsible AI integration in healthcare administration and clinical informatics. The proposed roadmap emphasizes data readiness, model validation, workflow integration, institutional accountability, post-deployment monitoring, and workforce readiness. The findings suggest that AI can contribute to more efficient, accessible, and patient-centered healthcare only when it is implemented within trustworthy medical informatics ecosystems supported by ethical governance, human oversight, and continuous evaluation.

## 1. Introduction

Healthcare systems worldwide are under increasing pressure from the growing burden of chronic diseases, population ageing, rising healthcare costs, workforce shortages, and persistent inequalities in access to care. These pressures have created a strong need for more efficient, sustainable, and data-driven models of healthcare delivery. At the same time, healthcare organizations are generating unprecedented volumes of data through electronic health records (EHRs), medical imaging, laboratory systems, genomics, administrative databases, telemedicine platforms, and wearable devices. These data sources provide important opportunities for improving healthcare delivery and administration, but their scale, heterogeneity, and complexity often exceed the capacity of traditional analytical approaches [1,2,3].

Artificial intelligence (AI) has emerged as a central technology in medical informatics and healthcare administration. By combining machine learning, deep learning, natural language processing (NLP), knowledge representation, predictive analytics, and more recently generative AI, intelligent systems can support pattern recognition, risk prediction, clinical decision support, workflow automation, resource allocation, and biomedical knowledge discovery [4,5,6,7]. However, the value of AI in healthcare does not depend only on algorithmic accuracy. It also depends on whether AI systems can be safely integrated into clinical information systems, administrative workflows, governance structures, and real-world decision-making processes.

One important milestone in the clinical adoption of AI was the validation of IDx-DR, an autonomous AI-based diagnostic system for detecting more-than-mild diabetic retinopathy in adults with diabetes who had not previously been diagnosed with diabetic retinopathy [8]. This example demonstrated that AI systems can move from experimental development toward regulated clinical use when they are supported by appropriate validation, safety evidence, and clearly defined indications for use. Since then, AI applications have expanded across diagnostic imaging, pathology, dermatology, cardiology, predictive analytics, drug discovery, hospital operations, and remote monitoring [9,10,11,12].

AI has also shown potential beyond diagnosis. In clinical decision support and precision medicine, machine learning models can analyze high-dimensional patient data to support risk stratification, treatment selection, and individualized care pathways [13,14,15]. In biomedical research, deep learning has accelerated molecular screening, antibiotic discovery, and protein structure prediction [16,17,18,19]. At the operational level, AI-driven predictive analytics can support patient flow management, discharge planning, hospital capacity prediction, remote monitoring, and administrative workflow optimization [20,21,22].

Despite these advances, real-world implementation of AI in healthcare remains uneven. Many AI tools demonstrate strong performance in controlled experimental settings but face important barriers when deployed in clinical and administrative environments. These barriers include poor data quality, fragmented EHR infrastructures, limited interoperability, dataset shift, algorithmic bias, lack of explainability, privacy and cybersecurity risks, limited clinician trust, and unclear regulatory accountability [1,23,24]. Recent reporting and evaluation guidelines such as CONSORT-AI, SPIRIT-AI, DECIDE-AI, and TRIPOD+AI emphasize that healthcare AI should be assessed not only in terms of predictive accuracy, but also in relation to clinical workflow integration, user interaction, safety monitoring, external validation, and real-world impact [25,26,27,28].

This review critically examines the role of AI in healthcare administration and clinical informatics. It focuses on four major domains: AI-enabled diagnosis and clinical decision support; treatment personalization and precision medicine; drug discovery and biomedical knowledge generation; and administrative optimization through predictive analytics, NLP, EHR-based systems, telemedicine, remote monitoring, and resource allocation. Rather than presenting AI as an isolated technological solution, this review examines the organizational, informatics, and governance conditions required for responsible implementation in real-world healthcare systems.

The main contributions of this review are threefold:

- It reframes AI adoption in healthcare administration from a medical informatics perspective, emphasizing integration into EHRs, clinical workflows, governance systems, and decision-making processes.
- It synthesizes evidence across diagnostic AI, treatment personalization, drug discovery, hospital operations, remote monitoring, public health, and mental health, while distinguishing technical potential from implementation readiness.
- It proposes a governance-oriented roadmap for responsible AI integration, focusing on interoperability, external validation, post-deployment monitoring, fairness, explainability, privacy, accountability, and workforce readiness.

**Figure 1:**
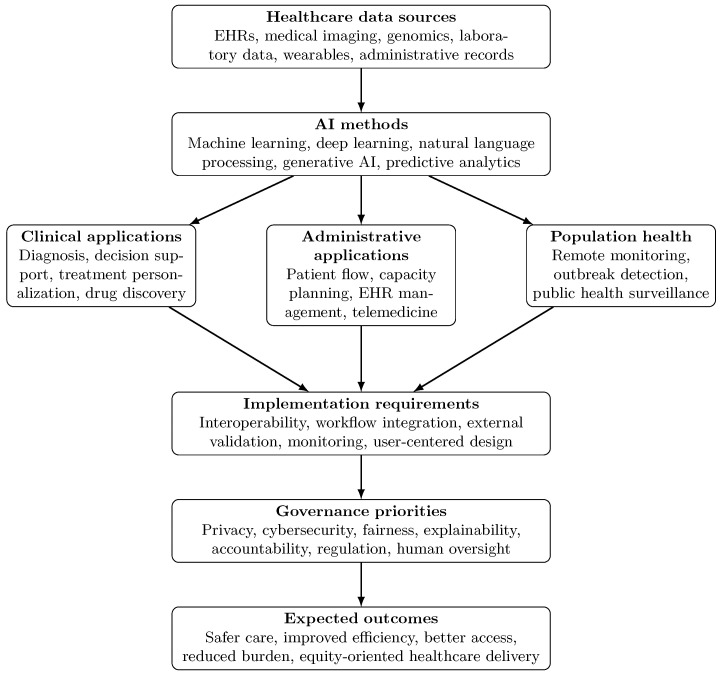
Conceptual framework for responsible AI integration in healthcare administration and clinical informatics.

Figure 1 presents the conceptual framework adopted in this review. It links healthcare data sources, AI methods, application domains, implementation requirements, governance priorities, and expected health system outcomes.

The remainder of this paper is organized as follows. Section 2 describes the review methodology and thematic synthesis approach. Section 3 presents the evolution of AI in healthcare and medical informatics. Section 4 reviews AI-enabled disease diagnosis across major clinical specialties. Section 5 discusses AI applications in healthcare administration and clinical informatics, including clinical decision support, drug discovery, hospital operations, EHR management, remote monitoring, public health, and mental health. Section 6 analyzes the main implementation challenges and ethical considerations. Section 7 proposes a governance roadmap for responsible AI integration. Section 8 discusses future directions and opportunities. Finally, Section 9 presents the limitations of the review, and Section 10 concludes the paper.

## 2. Review Methodology

This study was designed as a narrative critical review. The objective was not to conduct a quantitative meta-analysis, but to synthesize and critically discuss recent evidence on the role of artificial intelligence (AI) in healthcare administration and clinical informatics. The review focused on AI applications, implementation challenges, governance requirements, and future directions relevant to medical informatics systems.

The literature search was conducted across major scientific databases, including PubMed, Scopus, Web of Science, and ScienceDirect. Additional references were identified from the bibliographies of highly cited reviews, clinical evaluation studies, reporting guidelines, and policy documents. The search focused primarily on publications from 2018 to 2026, while earlier landmark studies were included when they provided important historical or conceptual foundations for AI in healthcare.

To improve transparency, the review process was organized into four steps: identification, screening, eligibility assessment, and thematic synthesis. During the identification stage, records were retrieved from the selected databases using combinations of keywords related to artificial intelligence, medical informatics, healthcare administration, clinical decision support, electronic health records, digital health, governance, and implementation. During the screening stage, titles and abstracts were reviewed to exclude publications that were unrelated to healthcare, purely technical without clinical or organizational relevance, or outside the scope of AI-enabled healthcare administration and clinical informatics. During the eligibility stage, full texts were examined to determine whether they contributed to at least one of the review domains or cross-cutting implementation themes. Finally, the included literature was synthesized thematically rather than statistically, because of the heterogeneity of study designs, healthcare settings, AI methods, and outcome measures.

The search strategy combined terms related to artificial intelligence, medical informatics, healthcare administration, and implementation. Search terms included combinations of the following keywords: “artificial intelligence”, “machine learning”, “deep learning”, “generative AI”, “foundation models”, “large language models”, “medical informatics”, “clinical decision support”, “electronic health records”, “healthcare administration”, “hospital management”, “healthcare operations”, “telemedicine”, “remote monitoring”, “AI governance”, “algorithmic bias”, “explainability”, and “healthcare ethics”.

Studies were considered eligible if they addressed at least one of the following themes: AI-enabled diagnosis, clinical decision support, treatment personalization, precision medicine, drug discovery, electronic health record analytics, natural language processing, hospital operations, remote monitoring, public health surveillance, AI governance, or ethical and regulatory issues in healthcare AI. Priority was given to peer-reviewed articles, systematic reviews, meta-analyses, clinical validation studies, consensus guidelines, and publications in high-impact medical, biomedical informatics, and digital health journals.

Publications were excluded if they were not directly related to healthcare, focused only on technical algorithm development without clinical or organizational relevance, lacked sufficient methodological or conceptual detail, or did not contribute to the objectives of this review. Non-peer reviewed reports were used only when they came from recognized international or regulatory organizations and provided relevant guidance on governance, ethics, or implementation.

The selected literature was synthesized thematically. First, studies were grouped according to their main application area, including disease diagnosis, treatment personalization, drug discovery, healthcare administration, remote monitoring, public health, and mental health. Second, cross-cutting implementation issues were extracted, including data quality, interoperability, external validation, workflow integration, algorithmic bias, explainability, privacy, cybersecurity, accountability, and workforce readiness. Third, these themes were used to develop a governance-oriented roadmap for responsible AI integration in healthcare administration and clinical informatics.

Because this article is a narrative critical review, it does not aim to provide an exhaustive quantitative synthesis of all available studies. Instead, it aims to identify major developments, recurring implementation barriers, and governance priorities that are relevant for the responsible deployment of AI in healthcare systems.

Table 1 summarizes the methodological approach used to identify, select, and synthesize the literature included in this narrative critical review.

## 3. Evolution of AI in Healthcare and Medical Informatics

The development of artificial intelligence in healthcare has followed a progressive transition from rule-based expert systems to data-driven, multimodal, and workflow-integrated medical informatics applications. This evolution reflects both technological progress and changing expectations regarding how computational tools should support clinical and administrative decision-making.

This evolution is also closely connected to the broader development of health informatics as an interdisciplinary field. Health informatics includes multiple subdomains, such as medical informatics, clinical informatics, public health informatics, nursing informatics, pharmacy informatics, bioinformatics, and primary care informatics. Recent reviews emphasize that technological trends, including AI, machine learning, automation, and advanced data integration, increasingly shape these subdomains and require closer integration between clinical expertise, information systems, and organizational processes [29].

Early applications of AI in medicine were mainly based on rule-based expert systems. Systems such as MYCIN and DXplain relied on manually encoded medical knowledge and explicit decision rules to support diagnostic reasoning and therapeutic recommendations. Although these systems were pioneering, their real-world impact remained limited because they were difficult to maintain, hard to scale, and insufficiently adaptable to evolving clinical knowledge and heterogeneous patient populations [30]. These early limitations highlighted a challenge that remains central to medical informatics: AI tools must be clinically useful, continuously updateable, and integrated into real-world health information systems rather than functioning as isolated decision tools.

During the 1990s and early 2000s, statistical learning and machine learning approaches became increasingly important in clinical prediction and decision support. Methods such as logistic regression, support vector machines, decision trees, random forests, and ensemble learning enabled more data-driven approaches to diagnosis, prognosis, risk stratification, and clinical scoring. Compared with rule-based systems, these methods were more adaptable to empirical data and could improve prediction performance in several clinical contexts. However, they often required extensive feature engineering and remained limited when applied to unstructured data, such as free-text clinical notes, medical images, and high-dimensional biomedical data.

The emergence of deep learning in the 2010s marked a major shift in healthcare AI. Deep neural networks, particularly convolutional neural networks and recurrent neural networks, enabled automated feature extraction from complex data modalities, including medical imaging, electronic health records, biosignals, pathology slides, and clinical text [3,31]. This period was associated with rapid progress in image-rich specialties such as radiology, dermatology, ophthalmology, pathology, and cardiology. Several systematic reviews and meta-analyses showed that deep learning systems can achieve high diagnostic performance in selected settings, while also emphasizing limitations related to external validation, reporting quality, bias, and prospective clinical evaluation [9,10].

More recently, foundation models and generative AI have expanded the scope of healthcare AI beyond task-specific prediction. Foundation models are trained on large and heterogeneous datasets and can be adapted to multiple downstream medical tasks, including diagnosis, documentation, clinical summarization, image interpretation, patient-facing communication, and decision support [32]. Large language models have demonstrated the ability to encode medical knowledge and answer clinical questions, but their use in healthcare also raises concerns about factual reliability, reasoning errors, bias, hallucinations, privacy, and unsafe recommendations [33]. These developments are particularly relevant to medical informatics because they shift attention from isolated AI models toward general-purpose systems that may interact with electronic health records, clinical workflows, patient portals, and administrative processes.

This historical evolution shows that the main challenge in healthcare AI has also changed. The question is no longer only whether AI can achieve high predictive performance in controlled datasets, but whether it can be safely and responsibly implemented within medical informatics systems. This requires robust validation, interoperable data infrastructures, explainable and auditable models, privacy-preserving governance, clinician involvement, continuous post-deployment monitoring, and equity-oriented evaluation.

Table 2 summarizes the evolution of AI in healthcare and medical informatics, from rule-based expert systems to data-driven, multimodal, and workflow-integrated applications.

## 4. AI-Enabled Disease Diagnosis

Accurate and timely diagnosis is a fundamental requirement for effective healthcare delivery. Diagnostic delays, misdiagnosis, and unequal access to specialist expertise can contribute to avoidable morbidity, mortality, and healthcare costs. In many health systems, diagnostic performance still depends heavily on the availability of trained clinicians, imaging infrastructure, laboratory capacity, and timely access to patient information. These constraints are particularly important in resource-limited settings, where specialist services may be scarce and diagnostic delays may be more frequent.

Artificial intelligence has become an important component of diagnostic medical informatics. Machine learning and deep learning models can analyze high-dimensional data from medical images, pathology slides, electrocardiograms, laboratory results, and electronic health records. By detecting complex patterns that may be difficult to identify through manual interpretation alone, AI systems can support earlier detection, reduce inter-observer variability, and improve diagnostic workflow efficiency [6,9,10]. However, diagnostic AI should not be viewed as an isolated algorithmic tool. Its clinical value depends on external validation, integration into clinical information systems, human oversight, transparency, and continuous monitoring after deployment.

### 4.1. AI in Medical Imaging

Medical imaging has been one of the most active areas for diagnostic AI, because radiology and screening programs generate large volumes of structured image data. Deep learning models have been applied to chest radiographs, computed tomography, magnetic resonance imaging, and mammography for the detection of clinically relevant abnormalities such as pneumonia, lung nodules, fractures, intracranial hemorrhage, and cancer-related findings [9,11].

In lung cancer screening, Ardila et al. developed a three-dimensional deep learning system for low-dose chest computed tomography. The model used current and prior CT volumes to estimate lung cancer risk and showed strong performance in both internal and independent validation settings [34]. Such studies illustrate the potential of AI to support radiologists in high-volume screening programs. Nevertheless, safe deployment requires evaluation across different patient populations, imaging devices, acquisition protocols, and institutional workflows.

In breast cancer screening, McKinney et al. evaluated an AI system for mammography across international datasets and reported performance comparable to expert readers, with reductions in false positives and false negatives [35]. In practice, AI may be used as a second reader, triage tool, or workload reduction mechanism. However, implementation in screening programs requires prospective evaluation, monitoring of interval cancers, analysis of radiologist interaction with AI outputs, and assessment of possible effects on equity and access.

Pathology is another important diagnostic domain for AI. Whole-slide imaging combined with deep learning enables automated detection of cancerous regions, tumor grading, and biomarker quantification. For example, deep learning algorithms have shown strong performance in detecting lymph node metastases in breast cancer pathology slides [36]. These tools can reduce inter-observer variability and accelerate diagnostic workflows, but clinical adoption depends on digital pathology infrastructure, image quality control, laboratory information system integration, and clear responsibility for final diagnostic decisions.

### 4.2. AI in Ophthalmology

Ophthalmology has been a leading field in the clinical adoption of diagnostic AI. Diabetic retinopathy screening is particularly suitable for AI because it relies on standardized retinal images and clearly defined referral criteria. The clinical validation of IDx-DR, an autonomous AI-based diagnostic system for detecting more-than-mild diabetic retinopathy, represented an important milestone in the translation of AI from research to regulated clinical use [8].

The importance of ophthalmology AI lies not only in diagnostic accuracy but also in service delivery. AI-based screening can extend access to diabetic retinopathy detection in primary care and underserved areas where ophthalmologists are not readily available. However, real-world deployment requires robust image quality control, clear referral pathways, patient safety procedures, and continuous monitoring of performance across demographic and clinical subgroups.

### 4.3. AI in Dermatology

Dermatology has also attracted substantial attention in diagnostic AI, particularly for skin lesion classification. Deep learning models trained on large image datasets have demonstrated performance comparable to dermatology experts in selected experimental settings, including the classification of benign and malignant skin lesions [12]. These systems may support triage, early detection, and access to preliminary assessment in areas where dermatology services are limited.

Recent systematic evidence suggests that AI algorithms can achieve strong performance in skin cancer diagnosis when compared with clinicians of different expertise levels [37]. However, the evidence also shows that performance may vary according to image quality, dataset composition, lesion type, clinical context, and level of external validation. In particular, insufficient representation of diverse skin tones and real-world imaging conditions may lead to biased performance and unequal diagnostic benefit. Dermatology AI should therefore be evaluated on diverse populations and implemented as a support tool within clinician-led care pathways rather than as a replacement for specialist judgment.

### 4.4. AI in Cardiology

AI is increasingly used in cardiology diagnostics, especially for the interpretation of electrocardiograms, echocardiography, and wearable sensor data. Deep learning models applied to ambulatory electrocardiograms have shown cardiologist-level performance for detecting and classifying cardiac arrhythmias [38]. Other AI-enabled ECG systems have been developed to detect conditions that are not always obvious on standard ECG interpretation, such as left ventricular systolic dysfunction [39]. These applications are important because ECGs are widely available, low-cost, and routinely used in clinical practice.

AI can also support echocardiography by assisting with image acquisition, segmentation, view classification, and functional assessment. In addition, machine learning models can integrate ECGs, imaging data, laboratory results, and EHR information to support risk prediction for atrial fibrillation, heart failure, sudden cardiac arrest, and other cardiovascular conditions. From a medical informatics perspective, the key challenge is not only model accuracy but also integration into clinical workflows, alert management, and avoidance of alarm fatigue.

Representative examples of AI applications in disease diagnosis are summarized in Table 3.

Recent real-world evidence also shows that diagnostic AI performance can vary substantially across institutions, patient populations, devices, and clinical workflows. This variability is partly explained by dataset shift, changes in case mix, differences in acquisition protocols, and local documentation practices. Empirical work on medical imaging data has shown that data drift monitoring is important for maintaining reliable performance after deployment and should not be replaced by aggregate performance monitoring alone [40]. These findings reinforce the need for local validation, prospective evaluation, and continuous post-deployment monitoring before diagnostic AI systems are scaled across healthcare settings.

Overall, AI has demonstrated substantial potential to improve diagnostic accuracy, efficiency, and access across several medical specialties. The strongest evidence currently comes from image-rich specialties such as radiology, ophthalmology, dermatology, pathology, and cardiology. However, widespread clinical adoption requires more than high retrospective performance. Diagnostic AI systems must demonstrate reliable performance across diverse clinical settings and be implemented within safe, monitored, and accountable clinical workflows.

## 5. AI Applications in Healthcare Administration and Clinical Informatics

AI applications in healthcare are no longer limited to diagnostic support. They increasingly contribute to clinical informatics, hospital administration, remote care, population health, and knowledge generation. From a medical informatics perspective, their value depends on the ability to transform heterogeneous healthcare data into actionable information that can support clinical decisions, administrative planning, patient engagement, and health system governance. These data include EHRs, medical images, genomic profiles, laboratory values, clinical notes, wearable sensor outputs, and administrative records [3,4,13].

This section reviews major AI application areas beyond diagnosis, with a particular focus on their relevance to healthcare administration and clinical informatics. The emphasis is placed not only on technical potential, but also on implementation readiness, workflow integration, data quality, and the conditions required for safe and effective deployment.

### 5.1. Clinical Decision Support and Treatment Personalization

Treatment personalization is one of the most important promises of AI in healthcare. Rather than relying only on standardized treatment protocols, AI systems can integrate patient-specific information such as clinical history, laboratory values, imaging findings, genomic data, lifestyle factors, and treatment response trajectories. This enables more precise risk stratification, individualized therapeutic recommendations, and adaptive care pathways [1,5,14].

#### 5.1.1. AI in Oncology and Targeted Therapies

Oncology is particularly suitable for AI-driven personalization because tumors differ widely in molecular characteristics, imaging patterns, treatment response, and prognosis. AI models can analyze tumor genomics, radiological images, digital pathology slides, and clinical records to identify molecular patterns and support treatment selection. By linking patient-specific tumor profiles with biomedical knowledge bases, clinical guidelines, and published evidence, AI-based systems may help oncologists identify more appropriate therapeutic options and reduce unnecessary toxicity [5,6].

One early and widely discussed example is IBM Watson for Oncology, which used natural language processing and machine learning to review clinical evidence and suggest treatment options. Its experience is better interpreted as a cautionary implementation case rather than as evidence of established clinical benefit. Reports on Watson for Oncology highlighted limitations related to transparency, local validation, evidence updating, workflow integration, and contextual adaptation to different healthcare settings [24]. This example illustrates that oncology AI systems should not be assessed only by their knowledge-base coverage or algorithmic design, but also by clinical utility, institutional fit, explainability, maintenance, and prospective evaluation.

#### 5.1.2. AI in Diabetes Management

Diabetes management requires continuous monitoring and timely therapeutic adjustment. Traditional finger-prick glucose testing provides intermittent measurements and may fail to capture rapid changes in glucose levels. Continuous glucose monitoring systems have transformed diabetes care by providing real-time glucose measurements throughout the day. When combined with AI algorithms, these data can be used to identify glucose trends, predict hyperglycemia or hypoglycemia, and provide personalized alerts to patients and clinicians [41,42].

Automated insulin delivery systems, often referred to as artificial pancreas or closed-loop systems, represent another important application of AI in diabetes care. These systems combine continuous glucose monitoring devices with insulin pumps and control algorithms that adjust insulin delivery in response to changing glucose levels. Evidence from systematic reviews and clinical studies suggests that closed-loop systems can improve glycemic control, reduce hypoglycemic episodes, and decrease patient burden [43,44]. Beyond individual care, AI can also support population-level analysis of diabetes data, enabling risk prediction, complication forecasting, and more personalized disease management strategies.

#### 5.1.3. Dosage Optimization and Adaptive Treatment

AI is also being explored for dosage optimization and adaptive treatment planning. Reinforcement learning is particularly relevant in this context because it can model sequential decision-making and update treatment strategies according to patient response over time. Unlike fixed dosing protocols, reinforcement learning models can theoretically learn dynamic policies that maximize clinical benefit while minimizing adverse events [15,45].

Potential applications include insulin adjustment, anticoagulant dosing, chemotherapy scheduling, sepsis management, and anesthetic control. However, the use of reinforcement learning in clinical care remains challenging. Models require high-quality longitudinal data, careful handling of confounding and missingness, causal interpretability, and prospective validation before deployment. For this reason, reinforcement learning should be viewed as a promising decision support approach rather than as a replacement for clinician judgment.

### 5.2. AI in Drug Discovery and Biomedical Knowledge Generation

Drug discovery is traditionally a long, expensive, and uncertain process. AI is increasingly used to accelerate several stages of this pipeline, including virtual screening, molecular design, protein structure prediction, drug repurposing, toxicity prediction, and clinical trial optimization [16,17,18]. Although drug discovery is not a hospital administration task in the narrow sense, it is relevant to clinical informatics because it connects biomedical knowledge generation with translational decision support and precision medicine.

In virtual screening, machine learning models can estimate the biological activity, toxicity, and pharmacological properties of large numbers of candidate compounds before laboratory testing. This can reduce the search space and prioritize molecules with higher potential for experimental validation. A notable example is the use of deep learning to identify candidate antimicrobial compounds, illustrating how AI can contribute to early-stage drug discovery [16].

Protein structure prediction represents another major contribution of AI to biomedical research. AlphaFold demonstrated highly accurate protein structure prediction and significantly advanced the ability to model protein targets relevant to disease mechanisms and drug design [18]. The AlphaFold Protein Structure Database has further expanded access to predicted protein structures at large scale, supporting research in molecular biology, pharmacology, and therapeutic development [19].

AI also supports drug repurposing, where existing drugs are evaluated for new therapeutic indications. During the COVID-19 pandemic, AI-supported analyses contributed to the identification of potential therapeutic candidates, such as baricitinib, for further clinical investigation [46]. Generative models have also been explored for designing novel molecules, reducing early-stage discovery time and enabling more targeted exploration of chemical space [17].

Examples of AI applications in drug discovery and biomedical knowledge generation are summarized in Table 4.

### 5.3. AI in Hospital Operations and Resource Allocation

Healthcare organizations are complex systems in which operational decisions directly affect patient outcomes, clinician workload, cost, and service quality. AI can support healthcare administration by improving patient flow, resource allocation, scheduling, capacity planning, discharge coordination, clinical documentation, and population-level monitoring. These applications are particularly relevant to medical informatics because they depend on the effective use of EHRs, administrative databases, real-time monitoring systems, and interoperable information infrastructures [14,22].

AI-driven predictive analytics can support hospital operations by forecasting admissions, estimating length of stay, predicting discharge readiness, and identifying risks of readmission. These tools can help administrators plan bed capacity, allocate staff, coordinate discharge processes, and reduce emergency department congestion. Recent reviews show that statistical and machine learning methods are increasingly used to predict patient discharges and support operational decision-making [20].

AI may also support scheduling systems by incorporating physician availability, patient urgency, equipment constraints, and expected procedure duration. Such systems can reduce delays, balance workload, and improve resource utilization. During public health crises, predictive models may help hospitals anticipate surges in demand and allocate intensive care beds, ventilators, and staff more effectively. Nevertheless, operational AI tools must be validated within the specific institutional context in which they are deployed, since hospital workflows, data quality, staffing models, and resource constraints vary widely.

Beyond patient flow and bed management, AI can also support operational functions related to inventory management and medical supply classification. For example, Durmuş et al. proposed a machine learning approach for efficient classification of medical supplies in healthcare inventory management [47]. Such applications are relevant to healthcare administration because supply classification, demand forecasting, and inventory optimization can affect cost control, service continuity, and the availability of critical medical materials. However, these tools should be evaluated in relation to procurement processes, local demand variability, supply chain disruptions, and institutional resource constraints.

Recent evidence also suggests that AI and predictive models are increasingly used in hospital settings, but their evaluation remains uneven. Nong et al. reported that predictive models are used in a substantial proportion of U.S. hospitals, including for inpatient risk prediction, high-risk outpatient identification, monitoring, treatment recommendation, billing automation, and scheduling. However, the study also emphasized the need for stronger evaluation of accuracy, bias, and governance in operational settings [48]. This supports the argument that healthcare administration AI should be evaluated not only by predictive performance, but also by fairness, safety, workflow impact, and institutional accountability.

### 5.4. EHR Management and Natural Language Processing

Electronic health records are central to modern healthcare, but much of their clinical value is embedded in unstructured text, including progress notes, discharge summaries, radiology reports, pathology reports, and referral letters. Natural language processing can extract clinically meaningful information from these free-text sources and transform it into structured data that can support clinical decision-making, research, billing, quality measurement, and population health analytics [3,13].

AI-powered documentation tools may also reduce administrative burden by supporting clinical note generation, speech recognition, coding assistance, and summarization of patient encounters. These systems can improve documentation efficiency, but they also raise important concerns regarding accuracy, hallucinated content, privacy, medico-legal accountability, and clinician oversight. For this reason, NLP-based EHR tools should be designed as human-in-the-loop systems, with transparent outputs and clear responsibility for final clinical documentation.

### 5.5. Remote Monitoring and Telemedicine

Remote monitoring and telemedicine have expanded rapidly with the use of wearable devices, mobile health platforms, and connected biosensors. These technologies continuously collect physiological data such as heart rate, activity levels, sleep patterns, oxygen saturation, and cardiac rhythm. When combined with AI algorithms, these data streams can support early detection of deterioration, identify cardiovascular anomalies, and enable proactive management of chronic diseases [21,49].

The Apple Heart Study provided large-scale evidence that smartwatch-based monitoring can identify irregular pulse patterns suggestive of atrial fibrillation and prompt further clinical evaluation [49,50]. At the same time, wearable-based monitoring must account for measurement error, device variability, false alerts, and unequal access to digital technologies [51]. In telemedicine, AI can transform remote patient data into actionable clinical summaries, support triage, and help clinicians prioritize patients who require urgent attention. These applications may improve continuity of care, but they require integration with EHRs and clear clinical escalation pathways.

Table 5 highlights representative AI-enabled remote monitoring and telemedicine applications.

### 5.6. AI in Public Health and Population-Level Informatics

AI has also become increasingly important in public health surveillance, epidemiology, and pandemic preparedness. By analyzing epidemiological data, human mobility patterns, travel data, digital traces, and healthcare utilization trends, AI and data-driven models can help detect emerging outbreaks, forecast disease spread, and estimate healthcare resource demand. During the COVID-19 pandemic, mobility and epidemiological models were used to understand transmission dynamics and evaluate the effects of control measures [52].

Early-warning systems can support global health security by identifying unusual disease patterns before they become widely recognized. For example, analysis of travel patterns and early reports of pneumonia of unknown origin helped estimate the potential for international spread during the early phase of the COVID-19 outbreak [53]. Beyond infectious disease surveillance, AI can support vaccination planning, antimicrobial resistance monitoring, climate-health risk assessment, and population health management. However, these applications require transparent data sources, careful interpretation, and governance mechanisms to prevent misuse of surveillance data.

### 5.7. AI in Mental Health and Digital Support Tools

AI is increasingly used in mental health to expand access to support, especially in settings where mental health professionals are scarce or where patients face barriers to in-person care. Conversational agents and chatbots can deliver structured psychological support, psychoeducation, and cognitive-behavioral therapy-based exercises through mobile interfaces. Examples such as Woebot and Wysa illustrate how conversational AI can provide low-threshold support and encourage self-reflection [54,55].

These tools may be useful for early support, symptom monitoring, and patient engagement, but they should not be considered substitutes for professional mental healthcare. Their use raises important concerns related to safety, crisis management, privacy, clinical effectiveness, and the quality of therapeutic interaction. NLP methods have also been explored for detecting signals of depression, anxiety, or suicidal ideation from clinical text, patient conversations, and digital behavior. Such applications require particularly strong ethical safeguards because of the sensitivity of mental health data and the potential consequences of false positives or false negatives.

Selected AI application areas in healthcare administration and clinical informatics are summarized in Table 6.

Overall, AI applications in healthcare are expanding from diagnosis and treatment support toward broader medical informatics functions, including healthcare administration, EHR management, population health, and remote care. The strongest contributions are likely to emerge when AI systems are linked to real clinical and administrative needs, evaluated in operational settings, and implemented through accountable organizational processes.

Table 7 provides a critical synthesis of the implementation maturity of selected AI application areas, highlighting their evidence level, main deployment barriers, and key medical informatics priorities.

## 6. Implementation Challenges and Ethical Considerations

Although artificial intelligence offers major opportunities for improving healthcare delivery and administration, its implementation in real-world clinical environments remains complex. In medical informatics, AI systems are not only technical tools; they become part of broader socio-technical systems that include clinicians, patients, electronic health records, institutional workflows, regulatory requirements, and governance structures. Therefore, the success of AI in healthcare depends on more than predictive accuracy. It also requires reliable data, interoperability, clinical validation, transparency, privacy protection, human oversight, and clear accountability.

### 6.1. Data Quality, Governance, and Interoperability

The performance and reliability of AI systems depend heavily on the quality, representativeness, and governance of the data used for training, validation, and deployment. Healthcare data are often fragmented across hospitals, laboratories, imaging platforms, registries, billing systems, and electronic health records. These data sources may differ in coding systems, terminology, documentation practices, completeness, and technical formats. As a result, an AI model trained in one institution may not perform reliably when transferred to another clinical environment.

Interoperability is therefore a central requirement for responsible AI implementation. Standards such as Fast Healthcare Interoperability Resources (FHIR) can support structured data exchange and improve the reuse of health data across systems. However, interoperability alone is not sufficient. AI development also requires consistent data definitions, transparent data provenance, high-quality annotation, and clear governance rules for access, sharing, and secondary use of clinical data. Recent work on health data standards emphasizes that data quality, representativeness, and documentation are essential for the safe development of AI-based healthcare applications [58,59].

Because healthcare data are highly sensitive, privacy and confidentiality must be protected throughout the AI lifecycle. De-identification, access control, secure storage, audit trails, and privacy-preserving analytics are necessary to reduce the risk of unauthorized use. At the same time, privacy protection must be balanced with the need for external validation and multi-institutional evaluation. This balance remains one of the major governance challenges in medical informatics.

### 6.2. Algorithmic Bias, Equity, and Generalizability

Algorithmic bias is one of the most important risks associated with AI in healthcare. Bias may arise from non-representative training data, historical inequalities in access to care, measurement errors, biased labels, or the use of inappropriate proxy variables. A widely discussed example showed that a population health management algorithm underestimated the needs of Black patients because it used healthcare spending as a proxy for health need. Since historical healthcare spending was lower for Black patients with similar illness burden, the algorithm reproduced and amplified existing inequities [60].

Similar risks exist in diagnostic AI. Dermatology models trained mainly on images from lighter skin tones may perform less reliably on darker skin tones. Models developed in high-income settings may also fail to generalize to low-resource environments because of differences in equipment, population characteristics, clinical practices, and disease prevalence. These examples show that high average accuracy can conceal poor performance in specific subgroups.

Equity-oriented evaluation should therefore include subgroup analysis, fairness audits, external validation, and continuous post-deployment monitoring. Dataset transparency is also essential. Recent consensus recommendations call for health datasets used in AI to be documented in ways that make their limitations, population coverage, and possible sources of bias visible to developers, clinicians, regulators, and patients [61].

### 6.3. Explainability, Trust, and Human Oversight

Many AI systems, especially deep learning and generative models, operate as complex black-box systems. Although they may achieve high predictive performance, their recommendations are not always easy for clinicians to interpret or verify. This lack of transparency can reduce trust, complicate clinical accountability, and make it difficult to detect errors or unsafe model behavior.

Explainable AI may help clinicians understand why a system produced a given recommendation, but current explainability methods remain imperfect. Some explanations may be unstable, overly simplified, or poorly aligned with clinical reasoning. Therefore, explainability should not be treated as a complete solution. AI systems should also communicate uncertainty, allow clinician review, and provide clear escalation mechanisms when predictions are uncertain or potentially unsafe.

A human-in-the-loop approach is particularly important for high-risk clinical decisions. In this model, AI supports clinicians by providing risk estimates, alerts, summaries, or recommendations, but final responsibility remains with qualified healthcare professionals. Ethical analyses of machine learning in healthcare emphasize that implementation must consider not only technical performance, but also human interaction, clinical context, and unintended consequences [62].

### 6.4. Workflow Integration and Clinical Safety

AI systems can fail not only because of poor algorithms, but also because of poor integration into clinical workflows. A model that performs well in a retrospective study may be ineffective or unsafe if it produces alerts at the wrong time, interrupts clinicians, increases documentation burden, or creates alert fatigue. For this reason, AI implementation should be evaluated as a socio-technical process involving users, clinical tasks, information systems, and organizational constraints.

Clinical safety requires prospective validation, usability testing, workflow analysis, and post-deployment monitoring. AI systems should be evaluated for their impact on patient outcomes, clinician workload, decision quality, care processes, and possible unintended effects. Reporting guidelines such as CONSORT-AI, SPIRIT-AI, and DECIDE-AI emphasize the need to describe AI interventions, user interaction, clinical integration, and safety monitoring in a transparent manner [25,26,27]. These guidelines are particularly relevant for medical informatics because they move evaluation beyond algorithmic accuracy toward real-world clinical impact.

### 6.5. Cybersecurity, Privacy, and Model Vulnerability

AI systems introduce new cybersecurity and privacy risks. Healthcare organizations already store large volumes of sensitive personal and clinical data and are frequent targets of cyberattacks. AI adds further risks, including data poisoning, adversarial inputs, model inversion, membership inference, and unauthorized extraction of training information. These threats can compromise patient privacy, distort model behavior, and reduce trust in AI-enabled clinical systems [63,64].

Privacy-preserving approaches, such as federated learning, secure multi-party computation, differential privacy, and strong access governance, may help reduce some of these risks. Federated learning is particularly relevant in healthcare because it can support collaborative model development across institutions without requiring the centralization of raw patient data [65,66]. In medical imaging and other data-intensive clinical domains, secure and privacy-preserving machine learning approaches can help balance model development with confidentiality, regulatory constraints, and institutional data governance requirements [67]. However, technical safeguards must be combined with institutional policies, audit trails, incident response plans, and continuous risk assessment. AI systems deployed in healthcare should therefore be treated as part of a broader cybersecurity and information governance ecosystem.

### 6.6. Regulatory Oversight and Accountability

Regulatory oversight remains a major challenge for AI-enabled healthcare. Traditional regulatory models were designed for relatively stable medical devices, whereas many AI systems may change over time through model updates, new data, or local recalibration. This creates difficulties for approval, monitoring, liability, and accountability. If an AI system contributes to an incorrect diagnosis or unsafe treatment recommendation, responsibility may be distributed among clinicians, healthcare institutions, software developers, vendors, and regulators.

Clear accountability frameworks are therefore needed. These should define responsibility for model validation, deployment, monitoring, updates, error management, and patient communication. Patients should also be informed when AI systems meaningfully influence diagnosis, treatment, prioritization, or access to services. The WHO guidance on ethics and governance of AI for health emphasizes that AI systems should protect autonomy, promote human well-being, ensure transparency, foster responsibility, and advance equity [23].

### 6.7. Workforce Readiness and the Digital Divide

AI adoption also raises workforce and infrastructure challenges. Healthcare professionals may fear that AI will replace clinical roles, particularly in fields such as radiology, pathology, and administrative services. In practice, AI is more likely to redistribute tasks and augment human work than to replace clinicians entirely. Nevertheless, healthcare workers need training in AI literacy, data interpretation, model limitations, uncertainty, and safe use of decision support tools. Recent policy work emphasizes that AI adoption in health systems requires workforce adaptation, new skills, and safeguards to ensure that technological change supports rather than undermines healthcare professionals [23,68].

The digital divide is another major concern. Institutions with strong data infrastructure, skilled personnel, and financial resources are more likely to benefit from AI than under-resourced settings. Without deliberate policy intervention, AI could widen existing inequalities between hospitals, regions, and countries. This risk is particularly important in low- and middle-income countries, where limited digital infrastructure, fragmented health information systems, and shortages of trained personnel may restrict the safe and equitable deployment of AI tools. Responsible AI adoption therefore requires investment in digital infrastructure, workforce training, local validation, and inclusive governance [23,57].

### 6.8. Summary of Key Challenges

Table 8 summarizes the main implementation challenges and ethical considerations associated with AI integration in healthcare administration and clinical informatics.

In summary, technical performance is necessary but insufficient for safe AI deployment in healthcare. Responsible implementation requires a socio-technical approach that connects data quality, clinical safety, organizational accountability, workforce readiness, and continuous oversight.

## 7. Governance Roadmap for Responsible AI Integration

The challenges discussed in the previous section show that responsible AI integration in healthcare cannot be achieved through technical performance alone. AI systems must be governed across their full lifecycle, from data preparation and model development to clinical deployment, monitoring, and continuous improvement. In healthcare administration and clinical informatics, governance should ensure that AI systems are safe, equitable, transparent, clinically useful, and aligned with institutional workflows and patient interests.

This section proposes a governance-oriented roadmap for responsible AI integration in healthcare administration and clinical informatics. The roadmap is organized around six stages: data readiness, model validation, workflow integration, governance and accountability, deployment monitoring, and workforce readiness. These stages are intended to guide healthcare organizations, researchers, policymakers, and technology developers in moving from experimental AI applications toward trustworthy real-world deployment.

The proposed roadmap is intended to operationalize established AI governance and risk-management principles within healthcare administration and clinical informatics. Its emphasis on transparency, accountability, equity, human oversight, and safety is consistent with the WHO guidance on ethics and governance of AI for health [23]. Its lifecycle structure also aligns with the NIST AI Risk Management Framework, particularly the functions of governing, mapping, measuring, and managing AI-related risks [69]. For adaptive or AI-enabled medical device software, the roadmap is also consistent with the FDA approach to predetermined change control plans, which emphasizes planned modifications, validation methodology, implementation procedures, and impact assessment [70]. Therefore, the roadmap should be understood not only as a conceptual framework, but also as a practical lifecycle structure for planning, deploying, monitoring, and updating AI systems in healthcare organizations.

### 7.1. Stage 1: Data Readiness and Interoperability

The first stage concerns the readiness of healthcare data for AI development and deployment. AI systems require data that are accurate, representative, well-documented, and clinically meaningful. Before model development, healthcare organizations should assess data completeness, coding consistency, missingness, provenance, population coverage, and potential sources of bias. This is particularly important because healthcare data are often fragmented across electronic health records, imaging systems, laboratory platforms, registries, and administrative databases.

Interoperability should also be addressed at this stage. Standards such as FHIR can support structured data exchange and improve the ability to reuse clinical data across institutions. However, interoperability should be combined with semantic consistency, transparent data documentation, and appropriate governance for data access and secondary use. Data readiness is therefore not only a technical requirement, but also a prerequisite for fairness, safety, and reproducibility in AI-enabled healthcare.

### 7.2. Stage 2: Model Development and Validation

The second stage focuses on model development and validation. AI models should be trained using data that are appropriate for the intended clinical or administrative use case. During development, model performance should be evaluated using clinically relevant metrics, calibration analysis, subgroup performance assessment, and robustness testing. Aggregate performance alone is insufficient, because a model may perform well overall while producing unsafe or biased predictions for specific patient groups.

External validation is essential before deployment. Models should be tested on data from institutions, populations, and time periods that differ from the training environment. This helps assess generalizability and reduces the risk of poor performance caused by dataset shift. For high-risk applications, prospective validation should be conducted before routine clinical use. Reporting guidelines such as TRIPOD+AI, CONSORT-AI, SPIRIT-AI, and DECIDE-AI provide useful guidance for transparent evaluation and reporting of AI-based clinical prediction and decision support systems [25,26,27,28].

### 7.3. Stage 3: Workflow Integration and Human Oversight

The third stage concerns integration into clinical and administrative workflows. Even a technically accurate AI model may fail if it is introduced without considering how clinicians, administrators, and patients interact with it. AI systems should therefore be designed around real clinical tasks, information needs, decision points, and user responsibilities.

Workflow integration should include usability testing, alert design, definition of escalation pathways, and assessment of clinician workload. AI outputs should be presented in a way that supports decision-making without creating unnecessary interruptions or alert fatigue. For high-stakes decisions, human oversight should be explicit. The role of AI should be to support qualified professionals, not to remove clinical responsibility or replace contextual judgment.

### 7.4. Stage 4: Governance, Accountability, and Patient Communication

The fourth stage addresses institutional governance and accountability. Healthcare organizations should define who is responsible for approving, deploying, monitoring, updating, and retiring AI systems. Governance structures should include clinicians, health informaticians, data scientists, ethicists, legal experts, administrators, and patient representatives when appropriate.

Clear accountability is particularly important when AI systems influence diagnosis, treatment, prioritization, resource allocation, or access to care. Organizations should define procedures for error reporting, incident review, model updates, and communication with affected stakeholders. Patients should also be informed when AI meaningfully contributes to decisions about their care, especially in high-impact clinical or administrative contexts. This aligns with broader ethical principles of transparency, autonomy, responsibility, and equity in AI for health [23].

### 7.5. Stage 5: Post-Deployment Monitoring and Continuous Evaluation

The fifth stage concerns post-deployment monitoring. AI systems may degrade over time because of changes in patient populations, clinical practice, coding patterns, equipment, disease prevalence, or organizational workflows. This phenomenon, often referred to as model drift or dataset shift, can reduce safety and reliability if not detected.

Healthcare organizations should monitor AI systems continuously after deployment. Monitoring should include predictive performance, calibration, subgroup performance, fairness metrics, alert burden, user interaction, clinical outcomes, and safety events. When performance degradation is detected, the system should be reviewed, recalibrated, retrained, restricted, or withdrawn depending on the level of risk. Post-deployment monitoring should therefore be treated as part of routine clinical quality assurance and information governance.

In operational terms, post-deployment monitoring should rely on predefined performance, safety, usability, and equity indicators. These may include discrimination, calibration, false-positive and false-negative rates, subgroup performance, missing-data patterns, alert volume, override rates, user response time, safety events, and outcome-related measures. Monitoring should also include data drift indicators, including changes in input data distributions, coding practices, patient case mix, device characteristics, and clinical workflow patterns. These indicators should be reviewed by an AI governance committee or an equivalent institutional oversight mechanism, with clear procedures for model recalibration, retraining, restriction, or withdrawal when performance degradation or safety concerns are detected.

### 7.6. Stage 6: Workforce Readiness and Organizational Learning

The final stage concerns workforce readiness and organizational learning. Responsible AI adoption requires clinicians, administrators, and health informatics professionals to understand the strengths and limitations of AI systems. Training should cover AI literacy, data quality, uncertainty, bias, explainability, privacy, and safe use of decision support tools.

Healthcare organizations should also develop mechanisms for feedback from users. Clinicians and administrators should be able to report errors, workflow problems, unsafe recommendations, and unexpected model behavior. This feedback should inform continuous improvement of AI systems and their governance processes. In this sense, responsible AI integration is not a one-time implementation event, but an ongoing learning process.

Table 9 summarizes the proposed governance roadmap for responsible AI integration in healthcare administration and clinical informatics.

Overall, this roadmap emphasizes that AI integration in healthcare should be managed as a lifecycle process. Each stage is connected to the others: weak data governance can undermine model validation, poor workflow integration can reduce clinical value, and lack of monitoring can allow unsafe performance drift to persist. A responsible governance framework should therefore combine technical evaluation, clinical oversight, organizational accountability, and continuous learning. Such an approach can help healthcare organizations move from isolated AI pilots toward safe, equitable, and scalable AI-enabled medical informatics systems.

## 8. Future Directions and Opportunities in AI-Driven Healthcare

AI-driven healthcare is moving from isolated algorithmic applications toward integrated medical informatics ecosystems. Future progress will depend not only on advances in machine learning, natural language processing, generative AI, multimodal modeling, robotics, edge computing, and remote monitoring, but also on the ability of healthcare organizations to implement these technologies safely, equitably, and sustainably. The next phase of AI adoption should therefore be understood as both a technical and organizational transformation.

### 8.1. Multimodal and Generalist Medical AI

One of the most important future directions is the development of multimodal AI systems capable of integrating heterogeneous clinical data, including medical images, pathology slides, genomic profiles, laboratory values, clinical notes, wearable sensor data, and electronic health records. Such systems may support more comprehensive diagnostic and prognostic reasoning by combining information that is usually interpreted separately in current clinical practice.

Foundation models and generalist medical AI may accelerate this transition. These models are designed to perform a wide range of medical tasks and may support diagnosis, documentation, image interpretation, clinical summarization, patient-facing communication, and administrative support [32,33]. However, current evidence remains largely concentrated on benchmark performance and selected evaluation settings. Their clinical utility in routine care is still uncertain and must be established through prospective, workflow-aware, and safety-focused evaluation. Large language and multimodal models also raise specific risks, including hallucinated content, factual inconsistency, automation bias, privacy leakage, unsafe self-care advice, unclear accountability, and difficulty in auditing model behavior. Recent WHO guidance on large multimodal models emphasizes the need for governance, transparency, safety, and human oversight before such systems are widely used in healthcare [57].

### 8.2. AI-Enabled Preventive and Predictive Healthcare

Future healthcare systems are expected to place greater emphasis on prevention, early intervention, and longitudinal patient monitoring. AI can support this shift by identifying high-risk patients, detecting early signs of disease progression, and enabling more timely interventions. Predictive models based on EHRs, imaging, genomics, and wearable data may help detect clinical deterioration, forecast hospital readmissions, identify patients at risk of complications, and support population health management [1,13].

In neurological, cardiovascular, metabolic, and oncological diseases, AI may contribute to earlier detection by identifying subtle patterns before symptoms become clinically obvious. Nevertheless, predictive systems must be evaluated carefully to avoid overdiagnosis, unnecessary anxiety, false alarms, and inequitable performance across patient groups. The success of predictive AI will therefore depend on calibration, subgroup evaluation, clinical workflow integration, and continuous post-deployment monitoring.

### 8.3. Precision Medicine and Adaptive Care Pathways

AI can further advance precision medicine by integrating clinical, molecular, imaging, behavioral, and environmental data to support individualized care pathways. In oncology, AI may help link tumor genomics, imaging phenotypes, pathology features, and treatment response data to support therapy selection and prognosis. In chronic diseases such as diabetes and cardiovascular conditions, AI-enabled monitoring systems may help adjust care plans based on real-time patient data and longitudinal trends.

Adaptive treatment strategies, including reinforcement learning-based decision support, represent another promising direction. These methods may help optimize treatment sequences, drug dosing, and follow-up strategies over time. However, their clinical deployment remains challenging because they require high-quality longitudinal data, careful causal interpretation, and prospective validation before they can safely influence treatment decisions [15,45].

### 8.4. AI for Biomedical Knowledge Generation

AI will continue to play an important role in drug discovery and biomedical research. Deep learning methods can accelerate virtual screening, toxicity prediction, molecular generation, protein structure prediction, and drug repurposing. The success of AlphaFold has shown how AI can transform biological knowledge generation by providing large-scale protein structure predictions that support target identification and therapeutic research [18,19].

Future opportunities include integrating AI with laboratory automation, knowledge graphs, multi-omics data, and clinical trial data. AI may also help identify suitable trial participants, improve trial design, monitor adverse events, and adapt recruitment strategies. However, these applications require strong governance to ensure transparency, reproducibility, and responsible translation from computational discovery to clinical benefit.

### 8.5. AI-Enabled Healthcare Operations and Administration

In healthcare administration, AI has the potential to improve operational efficiency by supporting bed management, appointment scheduling, patient flow, discharge planning, supply chain management, workforce allocation, and demand forecasting. Predictive analytics may help hospitals anticipate admission surges, optimize intensive care capacity, reduce waiting times, and improve resource utilization [20,22].

Future hospital information systems may increasingly combine real-time operational dashboards with AI-based forecasting and decision support. However, these systems must be evaluated not only by technical accuracy but also by their effects on clinician workload, administrative burden, patient safety, equity, and organizational performance. AI should therefore be embedded in human-centered workflows rather than introduced as an additional layer of complexity.

### 8.6. Remote Monitoring, Telemedicine, and Edge AI

The combination of AI, wearable devices, mobile health applications, and telemedicine platforms will support more continuous and decentralized models of care. Wearable sensors can generate real-time physiological data, while AI models can help identify abnormal trends, detect early deterioration, and support chronic disease management. Edge AI may allow some analyses to be performed locally on devices, reducing latency and dependence on cloud infrastructure.

These developments are particularly relevant for remote and underserved regions, where access to specialized care may be limited. AI-enabled mobile health units, teleconsultation platforms, and portable diagnostic tools may extend access to screening, follow-up, and preventive care. Nevertheless, these opportunities depend on digital infrastructure, connectivity, device reliability, data protection, and clear referral pathways.

### 8.7. Public Health, Pandemic Preparedness, and Global Collaboration

AI can also strengthen public health surveillance and pandemic preparedness by analyzing epidemiological data, mobility patterns, genomic surveillance data, and healthcare utilization trends. These tools may support outbreak detection, resource planning, vaccine distribution, and scenario modeling during public health emergencies [52,53].

At the global level, AI adoption will require collaboration between governments, healthcare institutions, researchers, technology developers, and international organizations. Without coordinated governance, AI benefits may remain concentrated in high-income settings, while low-resource health systems face additional risks from unvalidated or poorly adapted technologies. International standards and locally adapted implementation strategies will be necessary to ensure that AI contributes to global health equity rather than widening existing disparities.

These concerns are particularly important in low- and middle-income countries, where limited digital infrastructure, fragmented health information systems, shortages of trained personnel, and under-representation in AI training data may reduce the safety, usability, and fairness of imported AI tools. Global AI governance should therefore support local validation, capacity building, infrastructure investment, and context-sensitive implementation rather than assuming that models developed in high-resource settings can be directly transferred to all healthcare environments.

### 8.8. Education, Workforce Readiness, and Human-AI Collaboration

The future of AI in healthcare will also depend on workforce readiness. Clinicians, administrators, and public health professionals will need basic AI literacy, including an understanding of model limitations, bias, uncertainty, data quality, and appropriate use of decision support systems. Medical and health informatics education should therefore include AI governance, data ethics, workflow integration, and critical appraisal of algorithmic tools.

AI is unlikely to replace healthcare professionals entirely, but it will reshape tasks and responsibilities. The most sustainable model is human-AI collaboration, where AI supports documentation, triage, prediction, and decision support, while humans retain responsibility for contextual judgment, communication, empathy, and ethical decision-making.

### 8.9. Trustworthy AI Deployment

The long-term success of AI in healthcare will depend on trustworthy deployment. Future AI systems should be designed according to principles of fairness, transparency, usability, robustness, explainability, safety, and accountability. Recent guidance such as CONSORT-AI, SPIRIT-AI, DECIDE-AI, TRIPOD+AI, and FUTURE-AI reflects the increasing emphasis on evaluation, reporting, clinical integration, and post-deployment monitoring [25,26,27,28,71].

Recent implementation science literature further emphasizes that AI systems should be evaluated through staged real-world assessment rather than moved directly from retrospective validation to routine deployment. Longhurst et al. called for AI implementation science centers to evaluate clinical effectiveness in real-world settings [72]. Similarly, You et al. proposed a clinical-trials-informed framework for AI implementation, organized around safety, efficacy, effectiveness, and post-deployment monitoring [56]. These approaches support the view that responsible healthcare AI requires structured implementation pathways, continuous evaluation, and institutional governance.

Table 10 summarizes the main future directions and their implications for healthcare administration and clinical informatics.

In summary, the future of AI-driven healthcare lies not in automation alone, but in the responsible integration of intelligent systems into medical informatics infrastructures. AI can support earlier diagnosis, more personalized care, faster biomedical discovery, better hospital operations, and stronger public health preparedness. However, these benefits will only be realized if innovation is accompanied by rigorous validation, ethical governance, workforce training, interoperability, and equitable access.

## 9. Limitations of the Review

This review has several limitations that should be acknowledged. First, it was designed as a narrative critical review rather than a full systematic review or meta-analysis. Therefore, it does not aim to provide an exhaustive or quantitative synthesis of all available evidence on artificial intelligence in healthcare. Instead, it focuses on major developments, implementation challenges, and governance priorities that are particularly relevant to healthcare administration and clinical informatics.

Second, the field of AI-driven healthcare is evolving rapidly, especially in areas such as generative AI, foundation models, multimodal learning, and clinical decision support. As a result, some recent studies, regulatory developments, or implementation experiences may not have been captured. This limitation is particularly important because the evidence base for many emerging AI applications is still changing quickly.

Third, the studies included in the reviewed literature vary considerably in methodological quality. Many AI studies in healthcare rely on retrospective datasets, single-institution data, limited external validation, or simulated clinical settings. Consequently, strong algorithmic performance reported in controlled environments may not always translate into safe, effective, or equitable real-world implementation.

Fourth, this review covers a broad range of AI application areas, including diagnosis, treatment personalization, drug discovery, healthcare operations, remote monitoring, public health, and mental health. This broad scope allows a comprehensive medical informatics perspective, but it also limits the depth of analysis within each specific specialty or application domain. More focused reviews may be needed to evaluate individual areas in greater detail.

Finally, the proposed governance roadmap is conceptual and synthesis-based. It is intended to support responsible AI integration in healthcare administration and clinical informatics, but it has not yet been empirically tested across healthcare institutions. Future research should evaluate the roadmap in real-world settings and assess its usefulness for guiding AI implementation, monitoring, accountability, and organizational decision-making.

## 10. Conclusions

Artificial intelligence is increasingly shaping healthcare administration and clinical informatics by supporting diagnosis, treatment personalization, drug discovery, remote monitoring, public health surveillance, and hospital operations. The evidence reviewed in this paper shows that AI can improve the use of complex healthcare data, support clinical and administrative decision-making, reduce documentation and operational burden, and contribute to more proactive and patient-centered models of care. However, the value of AI in healthcare depends not only on algorithmic performance, but also on its safe integration into clinical information systems, organizational workflows, and governance structures.

This review highlights that AI implementation remains constrained by several technical, organizational, ethical, and regulatory challenges. These include fragmented data infrastructures, limited interoperability, poor data quality, algorithmic bias, lack of explainability, privacy and cybersecurity risks, unclear accountability, workforce readiness gaps, and insufficient real-world clinical validation. These barriers are particularly important in operational settings, where AI systems must interact with clinicians, patients, electronic health records, regulatory requirements, and institutional decision-making processes.

A central contribution of this review is the proposed governance-oriented roadmap for responsible AI integration in healthcare administration and clinical informatics. The roadmap emphasizes data readiness, model validation, workflow integration, institutional accountability, post-deployment monitoring, and workforce preparation. These elements are interdependent: weak data governance can undermine model reliability, poor workflow integration can reduce adoption and safety, and insufficient monitoring can allow performance drift or inequitable outcomes to persist.

The future of AI-driven healthcare should therefore not be framed simply as automation, but as the responsible integration of intelligent tools into trustworthy medical informatics ecosystems. AI can support earlier diagnosis, more personalized treatment, more efficient hospital operations, and stronger public health preparedness when it is designed and deployed with attention to safety, fairness, usability, accountability, and clinical relevance. Achieving this goal will require collaboration among clinicians, health informaticians, patients, policymakers, regulators, healthcare managers, and technology developers.

Particular attention should be given to low- and middle-income countries, where limited infrastructure, workforce constraints, and under-representation in training datasets may restrict the safe and equitable use of AI. Ensuring global benefit will require locally validated tools, capacity building, and governance mechanisms that account for different healthcare contexts.

In conclusion, AI has the potential to substantially improve healthcare accessibility, efficiency, and quality, but these benefits will only be realized if innovation is balanced with rigorous governance and ethical deployment. Future research should prioritize prospective evaluation, privacy-preserving data use, workforce readiness, and the inclusion of diverse populations and healthcare settings.

## Figures and Tables

**Table 1:** Summary of the review methodology.

Component	Description
Review type	Narrative critical review focused on AI in healthcare administration and clinical informatics
Databases searched	PubMed, Scopus, Web of Science, and ScienceDirect
Timeframe	Primarily 2018–2026, with earlier landmark studies included when relevant
Main themes	AI-enabled diagnosis, treatment personalization, drug discovery, EHR analytics, hospital operations, remote monitoring, public health, governance, and ethics
Inclusion priorities	Peer-reviewed articles, systematic reviews, meta-analyses, clinical validation studies, consensus guidelines, and policy documents
Synthesis approach	Thematic synthesis organized around application domains, implementation barriers, and governance priorities
Selection process	Four-step process: identification, title/abstract screening, full-text eligibility assessment, and thematic synthesis

**Table 2:** Historical development of AI Applications in healthcare and medical informatics.

Period	Main AI Approach	Healthcare Relevance
1970s–1980s	Rule-based expert systems	Diagnostic support based on manually encoded medical knowledge and explicit decision rules
1990s–2000s	Statistical machine learning	Risk prediction, clinical scoring, prognosis, and data-driven decision support
2010s	Deep learning	Automated feature extraction from medical imaging, EHRs, pathology slides, biosignals, and clinical text
2020s	Foundation models and generative AI	Clinical summarization, multimodal reasoning, decision support, documentation assistance, and workflow integration

**Table 3:** Examples of AI applications in disease diagnosis.

Specialty	AI Application	Representative Evidence	Potential Impact
Radiology	Lung cancer detection using CT scans	Deep learning CT model [34]	Screening support and reduction of diagnostic variability
Mammography	Breast cancer screening	International AI screening evaluation [35]	Second-reader support and workload reduction
Pathology	Tumor detection on whole-slide images	Deep learning pathology assessment [36]	Faster and more consistent diagnostic workflows
Ophthalmology	Diabetic retinopathy screening	IDx-DR clinical validation [8]	Autonomous screening and improved access
Dermatology	Skin lesion classification	Systematic review and meta-analysis [37]	Triage support and early detection
Cardiology	Arrhythmia detection using ECG	Deep learning ECG tools [38]	Early detection and preventive cardiology

**Table 4:** Examples of AI applications in drug discovery and biomedical knowledge generation.

Application Area	AI Contribution	Representative Evidence
Virtual screening	Predicts biological activity and toxicity before laboratory testing	Deep learning-based antimicrobial discovery [16]
Protein structure prediction	Predicts three-dimensional protein structures to support target discovery	AlphaFold and AlphaFold Protein Structure Database [18,19]
Drug repurposing	Identifies new therapeutic uses for existing drugs	AI-supported identification of baricitinib as a potential COVID-19 treatment [46]
Generative molecular design	Generates and optimizes candidate molecules for early-stage discovery	Deep learning-based molecular generation [17]

**Table 5:** Examples of AI-enabled remote monitoring and telemedicine applications.

Application Area	AI-Enabled Function	Potential Impact
Wearable cardiac monitoring	Detection of irregular pulse patterns and possible atrial fibrillation	Earlier clinical evaluation and preventive cardiovascular care [49]
Continuous physiological monitoring	Analysis of heart rate, activity, sleep, oxygen saturation, and trend changes	Early identification of deterioration and chronic disease management [21,51]
Telemedicine support	Transformation of remote patient data into actionable clinical information	Improved triage, continuity of care, and reduced unnecessary visits
Patient engagement	Personalized feedback, alerts, and self-management support	Improved patient participation in monitoring and prevention

**Table 6:** Summary of selected AI application areas in healthcare administration and clinical informatics.

Application Area	Main AI Contribution	Expected Outcome
Clinical decision support and treatment personalization	Integration of clinical, genomic, imaging, and behavioral data	More individualized risk assessment and care pathways
Drug discovery and biomedical knowledge generation	Virtual screening, molecular generation, protein structure prediction, and repurposing	Faster identification of promising therapeutic candidates
Hospital operations	Forecasting admissions, discharge readiness, length of stay, and resource needs	Improved capacity planning, patient flow, and workload management
EHR management and NLP	Extraction, summarization, and structuring of clinical information	Reduced documentation burden and improved data usability
Remote monitoring and telemedicine	Analysis of wearable and home-monitoring data	Earlier detection of deterioration and improved chronic disease follow-up
Public health and population-level informatics	Outbreak detection, forecasting, and population-level risk analysis	Improved preparedness and more timely public health response
Mental health and digital support tools	Conversational support, symptom tracking, and NLP-based risk detection	Expanded access to low-threshold psychological support

**Table 7:** Implementation maturity of selected AI application areas in healthcare.

Domain	Evidence Maturity	Main Implementation Barrier	Informatics Priority	Selected References
Medical imaging	High for selected tasks	External validation, dataset shift, and workflow integration	PACS/EHR integration and post-deployment monitoring	[9,10,40]
Clinical decision support	Moderate	Alert fatigue, clinician trust, and unclear accountability	Human-centered workflow design and prospective evaluation	[14,56]
EHR and NLP	Moderate to high	Data quality, hallucination risk, and interoperability	Standardization, auditability, and human oversight	[3,13,57]
Drug discovery	High scientific potential	Translation to clinical benefit	Reproducibility and data provenance	[16,18,19]
Hospital operations	Emerging	Local workflow variability and limited operational validation	Real-world implementation evaluation and resource optimization	[47,48]
Remote monitoring	Growing	False alerts, device variability, and digital divide	Device integration, escalation pathways, and equity monitoring	[49,51]
Mental health AI	Emerging	Safety, crisis management, and privacy	Clinical governance and risk protocols	[54,55]

**Table 8:** Key challenges for AI implementation in healthcare administration and clinical informatics.

Challenge	Main Risk	Required Response
Data quality and interoperability	Fragmented, incomplete, or inconsistent data may reduce model reliability	Data standards, provenance documentation, FHIR-based exchange, and data quality assessment
Algorithmic bias	Models may reproduce or amplify existing health inequalities	Representative datasets, subgroup analysis, fairness audits, and equity monitoring
Explainability and trust	Black-box recommendations may reduce clinician confidence and accountability	Uncertainty communication, interpretable outputs, and human oversight
Workflow integration	Poorly integrated AI may increase workload or create alert fatigue	Usability testing, workflow analysis, and human-centered implementation
Privacy and cybersecurity	AI systems may expose sensitive health data or be vulnerable to attacks	Privacy-preserving methods, access control, audit trails, and security monitoring
Regulation and accountability	Unclear responsibility may complicate safe deployment and error management	Clear governance frameworks, monitoring policies, and defined responsibilities
Workforce readiness and digital divide	Unequal infrastructure and limited AI literacy may widen disparities	Training, inclusive implementation, local validation, and infrastructure investment

**Table 9:** Governance roadmap for responsible AI integration in healthcare administration and clinical informatics.

Stage	Key Actions	Expected Contribution
Data readiness and interoperability	Assess data quality, completeness, provenance, representativeness, coding consistency, and interoperability	Improves reliability, reduces bias, and supports reuse of clinical data
Model development and validation	Conduct internal validation, external validation, calibration, robustness testing, and subgroup analysis	Ensures model safety, generalizability, and fairness across settings and populations
Workflow integration and human oversight	Perform usability testing, define decision points, design alerts, and clarify human responsibility	Improves adoption, reduces alert fatigue, and supports safe clinical decision-making
Governance and accountability	Define approval processes, accountability structures, patient communication procedures, and incident reporting	Strengthens transparency, responsibility, and regulatory alignment
Post-deployment monitoring	Monitor model drift, calibration, subgroup performance, false-positive and false-negative rates, alert burden, override rates, safety events, user interaction, and clinical impact	Supports continuous quality assurance, early risk detection, and timely model recalibration or withdrawal
Workforce readiness and organizational learning	Train clinicians and administrators, collect user feedback, and update governance processes	Promotes responsible human-AI collaboration and sustainable implementation

**Table 10:** Future directions for AI-driven healthcare.

Future Direction	Expected Contribution	Key Requirement
Multimodal and generalist AI	Integrates imaging, EHRs, genomics, text, and wearable data for broader clinical reasoning	External validation, transparency, and safeguards against unsafe outputs
Predictive and preventive care	Supports early risk detection, deterioration prediction, and population health management	Calibration, subgroup evaluation, and post-deployment monitoring
Precision medicine	Supports individualized treatment selection and adaptive care pathways	High-quality longitudinal data and clinically interpretable recommendations
Biomedical knowledge generation	Accelerates molecular discovery, protein structure prediction, and drug repurposing	Reproducibility, data provenance, and responsible translation
Healthcare operations	Improves patient flow, capacity planning, discharge coordination, and resource allocation	Workflow integration and institutional validation
Remote monitoring and telemedicine	Enables decentralized care, early deterioration detection, and chronic disease follow-up	Device reliability, EHR integration, privacy protection, and escalation pathways
Public health surveillance	Supports outbreak detection, resource planning, and emergency response	Transparent data governance and international collaboration
Workforce readiness	Promotes safe human-AI collaboration and responsible system use	AI literacy, training, and organizational learning

## Data Availability

No new data were created or analyzed in this study.
